# Alterations in Adiponectin, Leptin, Resistin, Testosterone, and Cortisol across Eleven Weeks of Training among Division One Collegiate Throwers: A Preliminary Study

**DOI:** 10.3390/jfmk5020044

**Published:** 2020-06-19

**Authors:** W. Guy Hornsby, G. Gregory Haff, Dylan G. Suarez, Michael W. Ramsey, N. Travis Triplett, Justin P. Hardee, Margaret E. Stone, Michael H. Stone

**Affiliations:** 1 College of Physical Activity and Sport Sciences, West Virginia University, Morgantown, WV 26505, USA; 2 School of Exercise, Biomedical and Health Sciences, Edith Cowan University, Joondalup 6027, Australia; g.haff@ecu.edu.au; 3 Center of Excellence for Sport Science and Coach Education, East Tennessee State University, Johnson City, TN 37614, USA; dylangsuarez@gmail.com (D.G.S.); RAMSEYM@mail.etsu.edu (M.W.R.); STONEME@mail.etsu.edu (M.E.S.); stonem@etsu.edu (M.H.S.); 4 Department of Health and Exercise Science, Appalachian State University Boone, NC 28607, USA; triplttnt@appstate.edu; 5 Centre for Muscle Research, Department of Physiology, University of Melbourne, Victoria 3010, Australia; justin.hardee@unimelb.edu.au

**Keywords:** throwers, testosterone, cortisol, adipokine, biochemical markers, athlete monitoring

## Abstract

Cytokine and hormone concentrations can be linked to the manipulation of training variables and to subsequent alterations in performance. Subjects: Nine D-1 collegiate throwers and 4 control subjects participated in this preliminary and exploratory report. Methods: Hormone (testosterone (T) and cortisol (C)) and adipokine (adiponectin, leptin, and resistin) measurements were taken at weeks 1, 7, and 11 for the throwers and weeks 1 and 11 for the control group. The throwers participated in an 11-week periodized resistance training and throws program during the fall preparatory period. Volume load was recorded throughout the study. Results: Hormone values did not exhibit statistically significant changes across time; however, there were notable changes for C, the testosterone to cortisol ratio (T:C), and adiponectin. Conclusions: T:C was increased as volume load decreased, and adiponectin increased in concert with decreases in C and increases in the T:C, possibly suggesting a lesser degree of obesity-related inflammation and a higher degree of “fitness” and preparedness.

## 1. Introduction

Training is a process in which a stimulus is applied, and with proper planning and associated recovery, adaptations can then occur. The stressors of daily life and training are cumulative and can overwhelm the adaptive ability of the athlete resulting in symptoms related to poor fatigue management, such as excessive inflammation and certain hormonal responses [1]. In some instances, chronic fatigue can potentially lead to the development of overtraining syndrome, where prolonged periods of high volume or intense training begin to result in otherwise unexplained deteriorations in performance along with symptoms such as fatigue, depression, muscle and joint discomfort, reduced appetite, and disrupted sleep [1,2]. Therefore, in addition to providing a sufficient stimulus to disrupt homeostasis, fatigue management is of primary importance in producing optimum performance adaptations. Poor fatigue management that results in maladaptations is often reflected by changes in resting hormone and cytokine concentrations that are indicative of accumulated fatigue [1,2].

Fatigue is a necessary consequence of training. Indeed, fatigue, in some form and to some degree, is required to elicit adaptation, and thus is not simply always undesirable [3,4]. Grandou [4] describes important aspects of the time course for recovery in an attempt to help coaches better understand and utilize functional overreaching, a strategy in which performance and/or relevant performance-based adaptation(s) rebounds beyond baseline following a period of increased fatigue. In contrast, nonfunctional overreaching involves a fatigue state that either lasts longer than desired and/or only leads to an eventual return to baseline and thus no performance improvement or adaptation enhancement [3,4]. While nonfunctional overreaching is ideally avoided, it is acute and thus less severe than overtraining syndrome (OTS), which is a chronic issue that requires a substantial de-loading period simply to return to baseline [3,4]. Cadegiani and Kater [3] describe that the general hormone symptomology between overreaching (functional and nonfunctional) and OTS can be similar (e.g., increased cortisol); however, important differences can exist in the magnitude and time course. Therefore, the monitoring of biochemical markers may offer some insight into how an athlete is responding to the training process.

Hormone and cytokine concentrations have been linked to the manipulation of training variables and subsequent alterations in performance [5,6,7]. An athlete’s total training volume (work performed) has been shown to influence the athlete’s hormone and cytokine profile [1,8]. For example, alterations in the testosterone/cortisol ratio (T:C) have been associated with changes in training volume as well as physiological aspects such as lean body mass (LBM), fat content, and strength/power performance [5,8,9]. Thus, the T:C ratio is commonly referred to as a marker of an athlete’s preparedness [5,8,9]. When the T:C ratio is elevated, an athlete is better able to express their cumulative adaptations, as fitness is higher in comparison to fatigue [5]. Cytokine production is part of the adaptive process; however, markedly increased/excessive cytokine accumulation has been related to poor fatigue management and overtraining [1,9]. Thus, the T:C ratio in combination with cytokine responses may provide a composite indication of how the volume and intensity of training are affecting the athlete physiologically.

Chronic fatigue, as well as the overtraining syndrome, both resulting from poor fatigue management, have been associated with excessive cytokine production and inflammation [1,9]. The adipokines adiponectin, resistin, and leptin are cytokines that have been associated with a number of health-related conditions related to obesity-induced inflammation [10]. Resting adiponectin concentrations are generally inversely related to these inflammatory conditions, while leptin and resistin are positively associated [10]. However, adiponectin appears to be positively correlated with non-obesity-related inflammatory conditions [10] and inflammation in tissues such as the joint synovium and colonic epithelium. This indicates that adiponectin may be regulated in the opposite direction in tissue-specific versus obesity-associated inflammatory conditions [10]. These findings suggest that all inflammation is not the same or at least is produced as a result of differing mechanisms. Although adiponectin concentration has been shown to increase acutely as a result of resistance exercise among weight-trained athletes [11], studies comparing the effects of endurance or resistance training on resting adiponectin concentration have found little effect over 12 [12] and 16 weeks [13]. An increased concentration of adiponectin would be a potentially advantageous trend because adiponectin is generally associated with reductions in obesity-related inflammation [14]; on the other hand, it could be related to increased joint inflammation, which is a more negative outcome of training. Therefore, further study needs to be conducted to better understand the response of adiponectin in athletes throughout a training period or competitive season.

Interestingly, adiponectin has been shown to have an inverse relationship with resting cortisol concentrations [15]. Fallo [15] demonstrated that glucocorticoids inhibit adiponectin due to both exogenous administration to healthy subjects and endogenous cortisol hyperproduction [15]. Prolonged poor fatigue management, often associated with increased glucocorticoid production, and an increase in an athlete’s overtraining potential can accompanied by increased psychological stress [16]. Increased cortisol and low adiponectin concentrations may be associated with psychological manifestations accompanying accumulated training-induced fatigue. For example, lower adiponectin concentrations have been associated with increased susceptibility to depressive behaviors and impaired glucocorticoid-mediated negative feedback on the hypothalamic–pituitary axis [17], which is a process related to cumulative fatigue. Therefore, decreased cortisol and corresponding increased adiponectin concentrations may be indicative of lower psychological stress levels, but this is not yet well studied in athletic populations.

Adiponectin, leptin, and resistin are proteins (adipokines) that are synthesized and released from adipose tissue, with small amounts being produced in muscle [14,18] Often measured in obesity studies, these three cytokines have been linked to body fat content, inflammation, increased resting, and exercise-induced peripheral resistance as well as increased blood pressure. Adiponectin is believed to be cardioprotective in nature, producing increases in nitric oxide and vasodilatation, and it is generally associated with reduced inflammation [14] Resistin and lepin are directly related to fat mass and are atherogenic; they promote oxidative stress by decreasing nitric oxide production and promote inflammation [18,19,20].

Among sedentary populations, leptin and resistin have both been positively correlated with a wide range of adverse health outcomes [18,19,20]. However, among well-trained athletes [21] (sprinters, middle distance, and marathon runners), resistin has been shown to be elevated in comparison to controls, even though whole-body insulin sensitivity and lipid oxidation levels were higher [20]. From a speculative standpoint, it may be possible that the elevated concentrations of resistin and leptin noted among track athletes by Peresghin et al. [22] were a result of non-obesity-linked inflammation (e.g., perhaps training related). Two studies examining leptin response to resistance training [23,24] found no significant changes, while a study by Guadalupe-Grau et al. [25] found significant changes in serum leptin levels in females after 9 weeks of resistance training (no significant differences were found in the males). Varady et al. [26] investigated acute adipokine responses for males placed in different exercise groups (resistance exercise, resistance exercise plus running, running only). The 2 groups that involved resistance training demonstrated statistically significant increases in adiponectin and statistically significant decreases in resistin. Leptin did not change.

### Unique Aspects: Nature of the Report

This investigation was intended to be largely descriptive and exploratory in an effort to better understand biochemical alterations in a real world, athlete monitoring setting (high ecological validity). Due to low sample size and other factors (mix of males and females, control group being non-athletes, low in size, lack of clear parameters for adipokines in a sport/training context), the authors did not consider this work to be a traditional biochemical experiment but rather more in the nature and style of a scientific report and fitting of the growing interest in applied sport science work (e.g., sport scientists writing and submitting data collected with athletes under their care). The project involved several unique aspects:

- The athletes were highly resistance trained and possessed high strength levels. As evidence of this, all of the males produced a peak force over 6000 Newtons and the females produced a peak force over 4000 Newtons in the isometric clean-grip mid-thigh pull, which was carried out periodically throughout the study (weeks 1, 7, and 11). See Haff et al. [5] for methodology used.
- The training prescription was measured and reported in detail, including both the weight room work performed (sets and reps, exercises, prescribed intensities, volume load, and training intensity) as well as the athletes’ practice data (particularly unique was the monitoring of every throws practice). To the authors’ knowledge, for training studies on throwers, the inclusion of the number of throws has yet to be included along with weight room data. Indeed, a primary goal was for the ability of the training plan to be replicated; thus, the specific sets and reps and intensities provided for each training day.
- There is an extensive body of literature investigating the effects of training on testosterone and cortisol. Of note is a highly ecologically valid study recently conducted by Painter et al. [27] in which the T:C ratio was monitored across several months of training in D1 track and field athletes. However, there is a lack of research in athletes examining the potential concomitant changes to adipokine concentrations over a given training period (e.g., training study). Of particular interest (secondary outcome) was the potential usefulness of the adipokines measured throughout as a monitoring tool, thus the inclusion of commonly utilized biochemical markers (T and C) along with the more exploratory adipokines.

## 2. Materials and Methods

Testing was conducted as part of an ongoing athlete monitoring program. All testing took place in the Exercise and Sport Science Laboratory on the campus of East Tennessee State University in accordance with East Tennessee State University Institutional Review Board approval (IRB # 09-233s, 9/4/09). All subjects read and signed informed consent documents pertaining to all testing procedures.

### 2.1. Subjects

Nine D-1 collegiate throwers and four control subjects participated in the study. Anthropometric data are presented in Table 1. These athletes had a resistance training background ranging from 2 to 4 years at the collegiate level. The ability level of the throwers (6 male and 3 female) ranged from conference champions and contending National Collegiate Athletic Association (NCAA) Division I regional qualifiers to conference non-scorers. Throwing performance (taken from NCAA sanctioned meets) ranged from 10.98 to 16.9 m in the shot put and 12.03 to 18.6 m in the weight throw. The throwers were instructed not to change their dietary habits during the study. Additionally, based on questionnaire data, none of the female athletes reported taking oral contraceptives during the study. The control subjects (3 males and 1 female) were sedentary individuals and were instructed not to change their dietary habits and to remain sedentary throughout the study. It is worth mentioning that the control group was overfat and thus, this potentially influenced adipokine concentrations (e.g., elevated leptin).

### 2.2. Experimental Design

Prior to the initiation of the study, the throwers executed 4 weeks of moderately high volume, moderate training intensity (loading) resistance, conditioning, and throwing. This 4-week period consisted of 3 × 5 (1 × 5 down set) repetitions in the weight room using a 3-week increasing load and 1-week unloading scheme. Additionally, there was a moderate to high volume of throws, 2–3 days per week of 20–50 throws/day. Furthermore, throwing days (throughout the 11 weeks) were preceded with various warm-up/conditioning drills, including high knees, butt kicks, karaoka, and 3–5 short submaximal (70–90% effort) sprints). Throws were followed by mid-section exercises (basket hangs, candlesticks) and light stretching.

This study was viewed as a hypothesis-generating study of a longitudinal nature, analyzing physiological changes over 11 weeks of training in 9 D-1 collegiate throwers. Throwers executed a block periodized/programmed [28,29,30] throws and resistance training and conditioning program (e.g., various weighted medicine ball throws, sprints) that was structured and sequenced to enhance strength characteristics to potentially optimize performance for the upcoming indoor conference championships and produce an initial foundation for training for the outdoor season. Laboratory personnel observed and recorded all training for every weight room and throwing session. A series of three testing periods were implemented periodically during the study (weeks 1, 7, and 11) to measure total T, C, adiponectin, leptin, and resistin. The control group were measured at weeks 1 and 11 (no mid-testing) and underwent the same testing protocol as the throwers.

### 2.3. Procedures

Subject height was measured using a stadiometer (Detecto, Webb City, MO, USA) and recorded to the nearest centimeter. Body mass was determined using an electronic scale and was measured to the nearest 0.1 kg (BodPOD, COSMED USA, Chicago, IL). Body composition was assessed through the use of plethysmography (BodPOD, COSMED USA, Chicago, IL USA) [31].

Blood was collected by a trained phlebotomist from an antecubital vein using 21Gx 1−1/2” multiple sample needles and 8.5 mL (16 × 100 mm) clot activator blood collection tubes. Blood draws occurred first thing Monday morning after a (minimum) 12 h fast. The samples were allowed to clot and were then centrifuged to separate the serum. Then, the serum was pipetted into Eppendorf tubes and frozen in a −80 ºF freezer. All samples were analyzed in duplicate at the end of the study. Testosterone (T), cortisol (C), adiponectin, leptin, and resistin were measured by ELISA (DRG International, Mountainside, NJ, USA), intra assay CVs were <4.1%**.**

### 2.4. Testing/Training Protocol and Timeline

Multiple sources in the literature served as the foundation for the training prescription [30,32,33]. Although indoor track and field is a season, it is unlike many team sports in that every competition is not of equal importance. For this group of athletes, the primary competition of the indoor season is the conference championships. Thus, the thrower’s overall program was designed to peak the athletes for this event. Since the experiment only continued until the start of the indoor season, the study primarily involved the preparation period of training, which preceded the competitive season. The primary objective of this training period was to raise the work capacity and strength endurance levels of the athletes and ensure a reasonable level of preparedness prior to the indoor season.

The resistance training program was sequenced with a series of three 3–4 week concentrated loads (summated microcycles) of training. These concentrated loads were based on Plisk and Stone [32] and Stone et al. [33,34] and consist of a primary adaptation objective for a given period of time. This periodization method is similar in construction to “Block” and “Phase Potentiation” models of periodization/programming, which encompass several concentrated loads and periods of accumulation, transmutation, and realization [29]. The approach of this model is to take advantage of training after-effects that can potentiate adaptations in the subsequent phases of training [29,32,33].

The study began with a brief period of higher volume and less technical work with an emphasis on strength endurance, eventually shifting to a focus on strength and a slight increase in technical work. Table 2 shows the weekly set and repetition schemes, Table 3 shows the relative intensities for the target sets (warm-up sets are not shown, but all warm-up sets were recorded and used in volume load data analyses) and down sets, and Table 4 illustrates the exercises. Volume load was calculated as repetitions times load. Exercises were chosen in concert with the set/repetition scheme to achieve the goals and objectives of each concentrated load.

### 2.5. Statistical Analyses

Data were analyzed using the Statistical Package for the Social Sciences (SPSS) 17.0 (SPSS Inc., Chicago, IL, USA). Multiple (1 × 3) repeated measures analysis of variance (ANOVA) (with Mauchley’s sphericity) was used to determine if statistically significant differences existed between the measurement times for all throwers tested. Additionally, multiple (1 × 2) ANOVAs were used to determine if statistically significant differences existed between the measurement times for all control subjects tested. To control for potential sex differences, a between-subjects factor was included for the repeated measures ANOVA. The alpha criterion for determining statistical significance was set at *p* ≤ 0.05. Additional analyses were performed using effect size (eta squared)) and the coefficient of variation for the percent gain over time and 95% confidence intervals [35]. Additionally, when appropriate, Pearson’s r was utilized to assess relationships. Where applicable, data were allometrically scaled to help obviate body mass differences among individuals and between sexes using the following equation: absolute value/body mass ^(0.67)^ [34,36].

## 3. Results

**Figure 1:**
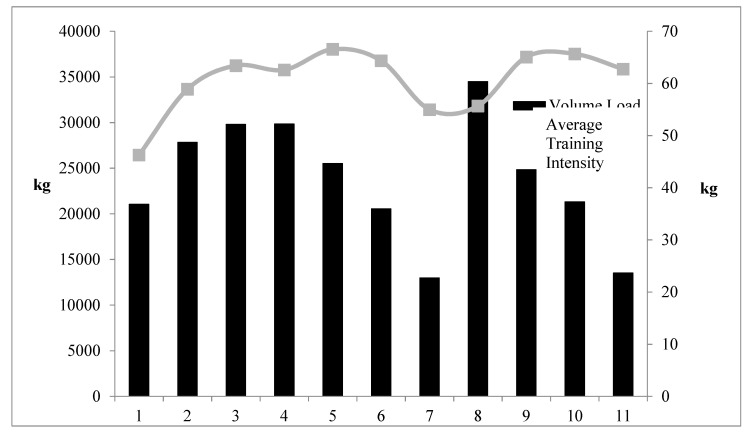
Throwers’ Weekly Resistance Training Volume Load and Average Weekly Training Intensity.

**Figure 2:**
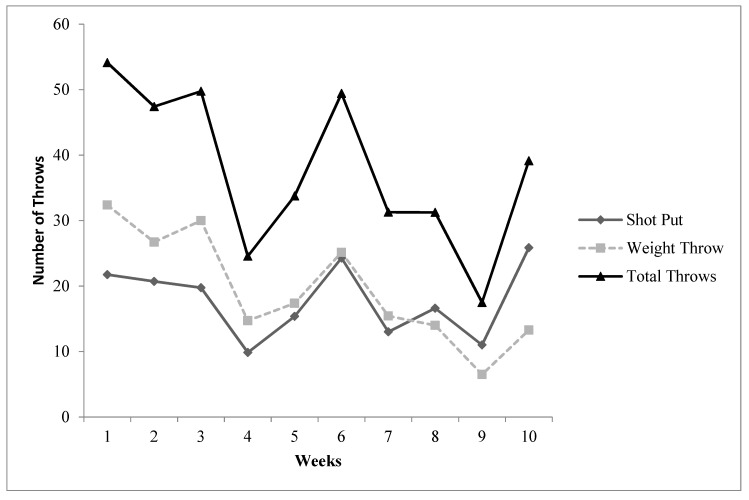
Throwers’ Weekly Throwing Volume.

**Figure 3:**
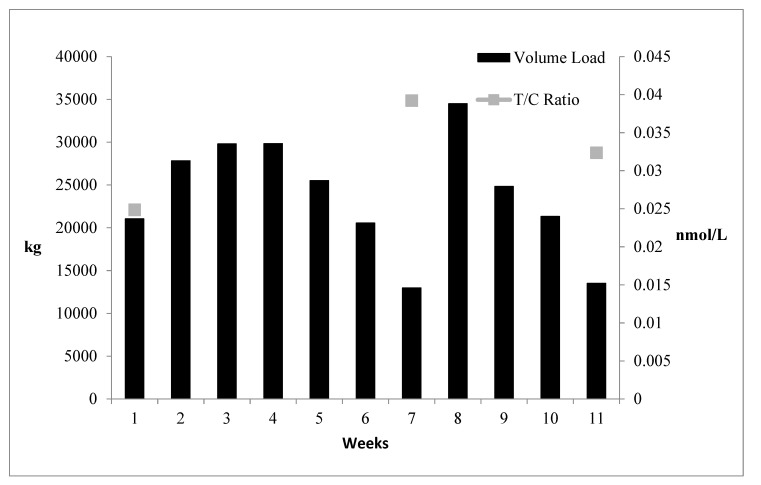
Throwers’ Volume Load and the Testosterone/Cortisol (T:C) Ratio.

**Figure 4:**
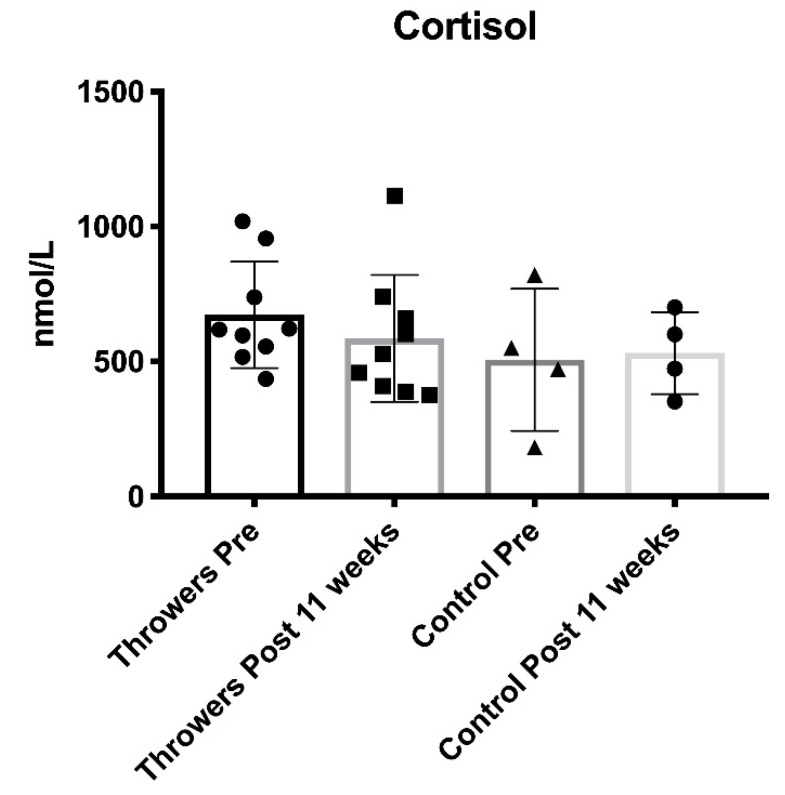
Cortisol Measures Pre and Post for the Throwers and Control Group.

**Figure 5:**
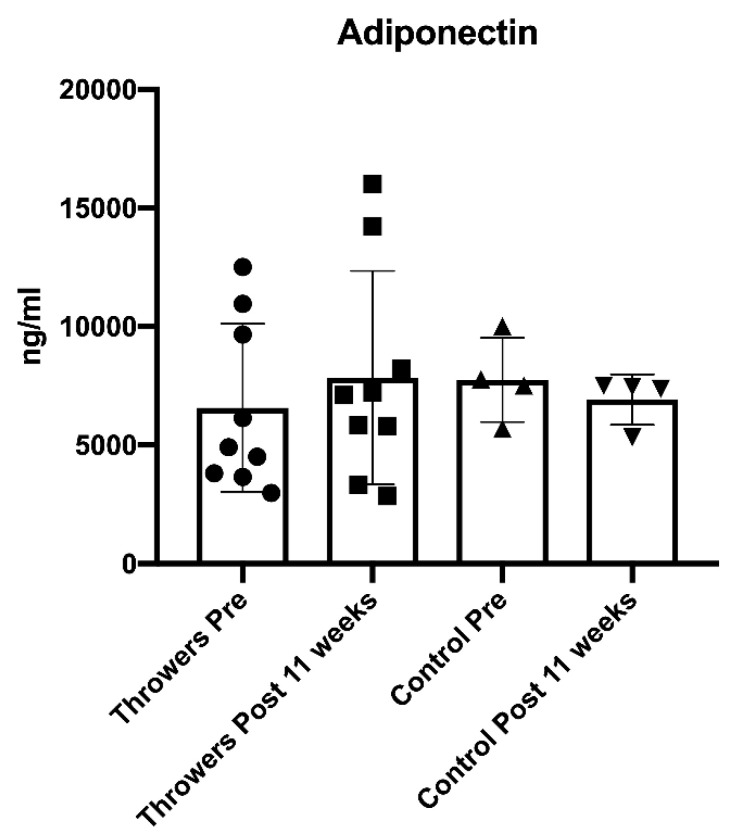
Adiponectin Measures Pre and Post for the Throwers and Control Group.

Training data are presented in Table 5 and Figure 1 (volume load and average training intensity) and Figure 2 (throwing data). No statistically significant differences were found between the control group and the throwers when analyzing resting T, C, adiponectin, leptin, and resistin blood concentrations. Additionally, there was no significant interaction effect for sex for any of the variables. Furthermore, neither the controls nor the throwers statistically changed body mass or body composition over the investigation. Group means, standard deviations, and statistical differences for the throwers’ hormone concentrations are presented in Table 6. Resting T, C, adiponectin, leptin, and resistin concentrations did not significantly change from T1 through T3. However, from a practical standpoint, it is important to note that based on effect size, %Δ (see Table 6), and the CV (see Table 7), T:C ratio (see Figure 3), C (see Figure 4), and adiponectin showed trends suggesting that training may have affected the resting concentrations. Interestingly, both C and adiponectin showed consistent alterations; C concentrations decreased over all 3 testing sessions (see Table 6 and Figure 4). Furthermore, adiponectin demonstrated increases across the 3 testing sessions (see Table 6 and Figure 5). An inverse relationship (r = −0.57) between the percent change in C and adiponectin was observed from T1 to T3.

## 4. Discussion

Based on the results of this investigation, it appears that the training program may have produced some beneficial physiological effects. These effects indicate a reasonable degree of fatigue management in that C decreased, and the T:C ratio was increased as the volume load decreased. The present observation indicates that adiponectin increased in concert with decreases in C and improvements in the T:C ratio. If increases in adiponectin are a positive outcome of a sound training protocol, these findings indicate a lesser degree of obesity-related inflammation and a higher degree of “fitness” and preparedness. Although the hormone and cytokine changes were relatively small, they may have been practically meaningful. Very small alterations in physiology and training performance can make large differences in competition [35]. It is also likely that these variables do not change to a large magnitude in the absence of very extreme training stimuli. Therefore, typical training periods where fatigue is effectively managed likely will not result in substantial hormonal or adipokine fluctuations.

No statistically significant changes were demonstrated in resting T, C, adiponectin, leptin, or resistin by the throwers or the control group. However, certain trends in the data may be associated with important alterations in the throwers’ physiology and performance. Three potential changes (C, T:C, and adiponectin) noted in the data are discussed below.

Cortisol is an anti-inflammatory and catabolic hormone. Increases in the concentration of C have been associated with relatively long-term (weeks) increased resistance training volume [8]. The steady decreases in C within this sample suggest that training stress was decreased at specific points in time. In support of this contention, measurements from weeks 7 and 11 corresponded to periods in which the volume load had been decreased, and training stress should have been at its lowest.

Of particular importance is the T:C ratio, as this is the ratio of a strong anabolic hormone and a strong catabolic hormone and is viewed as an anabolic index [5,37]. Thus, training-induced alterations in this ratio may reflect changes in the athlete’s physiology and preparedness. It has been suggested that only minimal changes in the T:C ratio may be necessary to effect performance alterations and preparedness for sport [5,27,37]. Before the present study was initiated, the throwers had just completed a moderate to a high volume of training lasting several weeks, which may partially explain the relatively low T:C ratio noted at T1. Subsequently, the T:C ratio was markedly higher compared to T1 at weeks 7 and 11 when training volume load had been reduced (Figure 3).

In the present study, there was a gradual increase in the throwers’ adiponectin levels (T1 = 6.573 ng/mL, T2 = 7.181 ng/mL, T3= 7.842 ng/mL), which appeared to parallel the reduction in C (T1 = 673 nmol/L, T2 = 612 nmol/L, T3 = 586 nmol/L). These findings perhaps suggest less fatigue and lower stress as training volume was reduced. It is particularly noteworthy that the increased serum adiponectin is perhaps reflective of a release of adiponectin caused by alterations in muscle production. This may have resulted in a “localized” protective mechanism that could counteract oxidative stress and resultant cellular damage [38]. While fluctuations occurred for the hormones and adipokines throughout the study, all 5 variables were considered to remain in “normal ranges”. Thus, this suggests that fatigue was properly managed.

It is worth noting that the female subjects’ menstrual cycles were not monitored. A recent study be Wyskida et al. [39] assessed menstrual cycle changes in 52 young, healthy, normal weight females measuring adiponectin, leptin and resistin (among other adipokines) at 2–4 days, 12–14 days, and days 24–26. Leptin (and vaspin) were affected by changes via menstrual cycle while resistin and adiponectin were not altered [39]. More specifically, leptin (and vaspin) demonstrated increases in both midcycle and luteal phases corresponding with changes in the secretion of testosterone, 17-OH progesterone, and insulin in the luteal phase [39]. Thus, a limiting factor of this is study is that for leptin, the menstrual cycle may have potentially influenced alterations for the female subjects. It is worth noting that none of the female athletes reported using hormonal contraceptives.

### 4.1. Practical Application

Coaches can use scientifically supported periodization models to manage fatigue and optimally direct training processes to optimally develop the preparedness of their athletes. The monitoring of biochemical markers may provide coaches some insight into how an athlete’s physiology changes throughout training. Some practical limitations for measuring biochemical markers include financial constraints and the understanding that values for biochemical markers are often not obtained immediately after a blood draw session. Due to the delay in receiving the values for a given marker(s), biochemical monitoring commonly is used for global reflection (e.g., responses across several phases of training). Thus, if a fatigue-related problem exists for an athlete, it may not be detected soon enough. Consequently, other monitoring tools are recommended for coaches to utilize (e.g., neuromuscular tests, questionnaires, etc.). Even in cases in which biochemical testing is far too impractical, detailed periodized/programming training studies using athletes, such as this one, can be useful for coaches that are determining what periodization/programming model they find most appropriate for their given situation. In this study, the periodized/programming training prescription appeared to soundly manage stress and fatigue as reflected by the small trends in certain biochemical markers such as C and adiponectin.

### 4.2. Relevant Contribution

In athletic populations and training environments, parameters (boundaries) for adipokines are not nearly as established as T and C; however, it is promising that all markers (both T and C and the three adipokines) stayed within “normal” ranges. While further study is certainly needed, it appears that adipokines are a potentially promising group of biochemical markers for the purposes of athlete monitoring. A major goal of athlete monitoring is to better understand both the training performed and the individual physiological response over time. Monitoring the training process of an athlete is best done with a constellation of markers versus a single marker and including the inflammatory-related hormone response may be another helpful piece of the overall athlete response puzzle.

## Figures and Tables

**Table 1:** Initial Subject Characteristics for Throwers and Controls.

Variable	Throwers (n = 9)	Controls (n = 4)
**Age (y)**	19.9 ± 1.1	23.6 ± 4.1
**Height (cm)**	181.8 ± 10.6	175.1 ± 7.4
**Weight (kg)**	105.5 ± 20.8	83.0 ± 13.6
**Body Fat (%)**	22.8 ± 5.8	28.0 ± 8.9

means and standard deviations.

**Table 2:** Training Phase and Corresponding Set and Repetition Scheme.

**Block 1**	**Strength Endurance**
Week 1	3 × 10
Week 2	3 × 5 (1 × 10)
Week 3	3 × 5 (1 × 10)
**Block 2**	**Strength Phase 1**
Week 4	5 × 5
Week 5	3 × 5 (1 × 5)
Week 6	3 × 5 (1 × 5)
Week 7	3 × 5 (1 × 5)
**Block 3**	**Strength Phase 2**
Week 8	3 × 10
Week 9	3 × 5 (1 × 5)
Week 10	3 × 3 (1 × 5)
Week 11	3 × 2 (1 × 5)

**Table 3:** Prescribed Relative Intensities in the Form of Percentage of Set-Rep Best.

Week	Monday	Wednesday	Thursday	Saturday
**B1**				
**1**	75–80%	70–75%	70–75%	80–85%
**2**	85–90%	75–80%	80–85%	80–85%
**3**	90–95%	80–85%	80–85%	80–85%
**B2**				
**4**	80–85%	70–75%	70–75%	80–85%
**5**	85–90%	70–75%	80–85%	80–85%
**6**	90–95%	80–85%	80–85%	80–85%
**7**	80–85%	80–85%	70–75%	70–75%
**B3**				
**8**	80–85%	70–75%	75–80%	80–85%
**9**	85–90%	75–80%	80–85%	80–85%
**10**	90–95%	80–85%	80–85%	80–85%
**11**	95–100%	80–85%	85–90%	80–85%

**Table 4:** Exercises across the Three Blocks of Training.

Block 1	Block 2	Block 3
**Monday**	**Monday**	**Monday**
AM	AM	AM
Squats	Squats	Squats
Press	Push Press	Push Jerk
PM	PM	PM
Bench Press	Incline Press	Incline Press
Dumbbell Front Raise	Dumbbell Front Raise	Dumbbell Front Raise
**Wednesday**	**Wednesday**	**Wednesday**
AM	AM	AM
Light Power Snatch	Light Power Snatch	Light Power Snatch
Clean Grip Shoulder Shrugs	Clean Grip Shoulder Shrugs	Clean Grip Shoulder Shrugs
Clean Grip Mid-Thigh Pulls	Clean Grip Mid-Thigh Pulls	Clean Grip Mid-Thigh Pulls
PM	PM	PM
Light Power Snatch	Light Power Snatch	Light Power Snatch
Clean Grip Shoulder Shrugs	Clean Grip Shoulder Shrugs	Clean Grip Shoulder Shrugs
Clean Grip Pulls from Knee	Cleans (1 target set)	Cleans (1 target set)
**Friday**	**Friday**	**Friday**
AM	AM	AM
Squats	Squats	Squats
Press	Push Press	Push Jerk
PM	PM	PM
Bench Press	Incline Press	Incline Press
Dumbbell Front Raise	Dumbbell Front Raise	Dumbbell Front Raise
**Saturday**	**Saturday**	**Saturday**
Light Power Snatch	Light Power Snatch	Light Power Snatch
Ball Throws	Ball Throws	Ball Throws
Pull Ups		

**Table 5:** Training Program Summary Data.

	Volume Load (kg)			Training Intensity (kg)		
**Weeks 1–7**	136,639.9	±	41,143.7	59.3	±	14.9
**Weeks 8–11**	94,159.2	±	28,389.6	62.3	±	15.0
**Weeks 1–11**	230,799.1	±	69,272.2	60.4	±	14.9

Volume Load: Wks 1–7 > wks 8–11 (*p* = 0.93, ES = 1.24).

**Table 6:** Hormone and Adipokine Data over Time for Throwers (Means +/− SD, Effect Size and %Δ).

	Testing 1	Testing 2	Testing 3	η^2^	%Δ Pre to Week 11
**Testosterone (nmol/L)**	14.6 ± 10.3	18.9 ± 15.7	14.9 ± 11.1	0.24	0.2
**Cortisol (nmol/L)**	673 ± 197	612 ± 265	586 ± 235	0.39	−12.9
**T:C ratio (nmol/L)**	0.025 ± 0.018	0.039 ± 0.036	0.032 ± 0.029	0.23	28
**Adiponectin (μg/mL)**	6.573 ± 3.539	7.181 ± 5.175	7.842 ± 4.501	0.29	19.3
**Leptin (ng/mL)**	19.877 ± 10.739	17.902 ± 16.363	20.851 ± 12.18	0.066	4.9
**Resistin (ng/mL)**	30.7 ± 12.3	37.9 ± 16.6	25.4 ± 14.4	0.48	−17.3

**Table 7:** Coefficient of Variance for Throwers.

Comparison Times	Variable	Coefficient of Variance			
		(%)	95% Confidence Interval		
T1 vs. T2	Testosterone	45.3	28.7	-	104.6
	Cortisol	18.3	12.0	-	38.0
	T:C Ratio	44.8	28.4	-	103.3
	Adiponectin	81.7	49.7	-	214.0
	Leptin	88.7	53.5	-	237.4
	Resistin	31.6	20.4	-	69.3
T1 vs. T3	Testosterone	39.3	25.1	-	88.8
	Cortisol	16.0	10.5	-	32.8
	T:C Ratio	37.5	24.0	-	84.1
	Adiponectin	35.8	23.0	-	79.8
	Leptin	49.1	31.0	-	115.1
	Resistin	35.6	22.8	-	79.2
T2 vs. T3	Testosterone	31.0	20.0	-	67.7
	Cortisol	20.4	13.3	-	42.6
	T:C Ratio	37.2	23.8	-	83.2
	Adoponectin	59.8	37.3	-	145.5
	Leptin	113.4	66.9	-	327.3
	Resistin	36.3	23.3	-	81.0
